# Circulating Protein Fragments of Cartilage and Connective Tissue Degradation Are Diagnostic and Prognostic Markers of Rheumatoid Arthritis and Ankylosing Spondylitis

**DOI:** 10.1371/journal.pone.0054504

**Published:** 2013-01-24

**Authors:** Anne C. Bay-Jensen, Stephanie Wichuk, Inger Byrjalsen, Diana J. Leeming, Nathalie Morency, Claus Christiansen, Morten A. Karsdal, Walter P. Maksymowych

**Affiliations:** 1 Rheumatology, Nordic Bioscience, Herlev, Denmark; 2 Division of Rheumatology, University of Alberta, Edmonton, Alberta, Canada; Keio University School of Medicine, Japan

## Abstract

Inflammation driven connective tissue turnover is key in rheumatic diseases, such as ankylosing spondylitis (AS). Few biomarkers are available for measuring disease prognosis or the efficacy of interventions applied in these tissue-related conditions. Type II collagen is the primary structural protein of cartilage and type III collagen of connective tissues, and obvious targets for the collagenalytic, which increase during tissue inflammation. The objective of the study was to investigate the diagnostic and prognostic utility of cartilage, C2M, and synovial, C3M, turnover biomarkers in AS. Serum samples were retrieved from patients suffering from AS (n = 103), RA (n = 47) and healthy controls (n = 56). AS progressors were defined as having new vertebral syndesmophytes or more that 3 unit change in mSASSS over a two-year period. Type II collagen degradation markers in serum were measured by the C2M ELISA, and type III collagen degradation by the C3M ELISA. Logistic regression and dichotomized decision tree were used to analyze the prognostic value of the markers individually or in combination. Both C2M and C3M levels were significantly higher in RA patients than in healthy controls (*p<0.0001*). Diagnostic utility was analyzed by ROC and areas under the curve (AUCs) were 72% and 89% for C2M and C3M, respectively. Both C2M and C3M, were significantly higher in serum samples from AS patient than from healthy controls (*p<0.0001*). The AUCs of C2M and C3M, respectively, were 70% and 81% for AS. A combination of C2M and C3M, dichotomized according to best cut-offs for individual markers, could correctly identify 80% of the progressors and 61% of the non-progressors. The present study is the first to show that specific biomarkers of cartilage and connective tissue degradation facilitate both diagnosis and prediction of progression of RA and AS.

## Introduction

Ankylosing spondylitis (AS) and rheumatoid arthritis (RA) are both chronic inflammatory arthropathies that are associated with excessive turnover of connective tissues, such as cartilage and ligaments, in and surrounding the affected joint [1]. RA is a systemic autoimmune disease that primarily attacks synovial joints leading to deterioration and loss of mobility. Moreover synovial inflammation and fibrosis are key events in the pathogenesis of RA [1]. Bone changes such as erosions, bone marrow lesions, and osteitis are also common pathological features of RA [2]. AS is also a systemic disease characterized by chronic inflammation of the sacroiliac joints, entheses, bone marrow, and structural lesions such as, syndesmophytes and joint ankylosis [3]–[5]. Accordingly, a common denominator of RA and AS is an elevated, inflammation-dependent turnover of connective tissue, specifically the extracellular matrix (ECM), in cartilage and synovium. ECM composition varies between connective tissues. The major ECM protein of cartilage is type II collagen, while type III collagen is the key protein of soft tissue (such as the synovium and entheses). Monitoring the turnover of these collagens may aid the understanding of the pathogenesis of RA and AS.

In pathological situations, such as RA and AS, inflammation disturbs the normal repair response leading to excessive remodeling and tissue turnover. The consequence of this heightened ECM remodeling is the release of a range of protein-degradation products generated by the proteases expressed locally in the pathologically affected area. The degradation fragments result in the exposure of *de novo* sites in the proteins, referred to as neo-epitopes. These protein degradation fragments may be specific for the tissue of origin and for the involved enzymes, and may consequently be used for the identification of molecular biochemical markers [6]. Recently, neo-epitope-based biochemical markers measured in serum have received increased attention for their diagnostic and prognostic potential in rheumatic diseases [7]. In slowly progressing diseases, such as osteoporosis and osteoarthritis (OA), bone resorption and cartilage degradation markers have been studied extensively [8].

It has been shown that cartilage degradation is highly elevated in RA patients, as measured by C-terminal telopeptide of type II collagen (CTX-II) in urine and *in situ* histological assessments [9]. These specific activities are precisely coordinated under physiological situations, with a specified sequence of events resulting in controlled tissue turnover.

Only a few serological biomarkers with diagnostic and prognostic properties are available for rheumatic diseases [10]. Most available biomarkers measure systemic and inflammatory factors, which do not reflect pathogenesis in the affected tissue. There is also a lack of sensitive biomarkers that can be used to predict those patients who will progress and those who will respond to treatment. One of the few examples of a prognostic biomarker is MMP3 measured in serum [11], which was elevated in patients who had an mSASSS change of more than 3 over a 3-year period. We recently developed two neo-epitope biomarkers C2M [12] and C3M [13], which directly measure tissue remodeling, and ultimately might be used to predict progression in inflammatory diseases. C2M is a serum biomarker that measures a matrix metalloproteinase (MMP)-generated neo-epitope of type II collagen and correlates with the severity of knee OA. C2M therefore reflects cartilage degradation [14]. C3M is a biomarker of soft tissue turnover associated with inflammation [15]–[17], which has been shown to be elevated in liver, skin and lung fibrosis. We recently reported that both biomarkers were highly elevated in AS and that the diagnostic utility for the combination of these two biomarkers was high (AUC 0.87, *p<0.0001*) [14]. The primary aims of the present study were to (i) to compare the diagnostic utility of these markers in AS versus RA and (ii) to investigate the prognostic potential of these novel connective tissue turnover markers in patients with AS.

## Methods

### Serum samples from healthy individuals and patients

Serum samples from controls were retrieved from two separate studies [12], [14]. Subjects were all lean (BMI<25 kg/m2), between 21 and 72 years of age, had no history of rheumatic or arthritic disease or treatment for such. All subjects felt healthy and had no reported pain or symptoms of any disease. All patients signed an informed consent. Baseline characteristics are shown in Table 1.

**Table 1:** Baseline demographics and clinical assessment scores for the healthy controls, RA and AS patients.

	Controls Mean (SD)	RA patients Mean (SD)	AS patients Mean (SD)
N	56	47	103
Mean age (SD), years	42.8 (10.5)	55.3 (12.4)	42.0 (13.6)
Number of Female/male	21/35	12/35	30/94
NSAID users, %	0	17	84
Disease duration, years	–	14.4 (10.7)	18.0 (11.9)
RF positive, %	–	86	0
Mean ESR (SD), mm/hour	–	40 (29)	22 (20)
CRP (mg/L)	–	33.5 (45.1)	14.2 (22.2)
BASDAI	–	–	5.6 (2.2)
mSASSS	–	–	14.2 (18.5)
DAS28	–	6.6 (1.2)	–
HAQ	–	1.9 (0.8)	–
TJC	–	18.2 (7.9)	–
SJC	–	13.5 (5.9)	–

Serum samples were collected from patients suffering from AS (n = 106). Of the 106 patients, 94 had 2-year radiographic follow-up. All patients had an established diagnosis of AS according to the modified New York Criteria [18]. Patients had received standard care, including physical therapy and treatment with a non-steroidal anti-inflammatory drug (NSAID), but were naïve to biologics at baseline. For progression analysis, patients were separated into two groups based on the presence (Yes/No) of a new syndesmophyte at 2-year follow-up. Bath AS Disease Activity Index (BASDAI) [19] and modified Stoke AS Spinal Score (mSASSS)[20] were recorded for each AS patient. Baseline characteristics are shown in Table 1.

Serum samples from RA patients (n = 47) were retrieved prior to the start of treatment with biologics. Patients are eligible for biologics in the Province of Alberta, Canada, if they had active disease despite treatment with methotrexate, a methotrexate combination with a second disease-modifying agent, or leflunomide. Baseline characteristics are shown in Table 1.

The retrieval of serum samples from healthy subjects and patients was approved by the Danish national Committee on Biomedical Research ethics (approval no KA 2006–0054), and by the Ethical board of University of Alberta, Edmonton, Canada. The study was conducted in the countries of residence of the authors, Denmark and Canada respectively.

### Biomarker assays

Cartilage degradation and connective tissue inflammation were measured in all serum samples, whether from controls or patients with AS or RA, using competitive enzyme-linked immune sorbent assays (ELISAs) for C2M [12] and C3M [15], respectively. The C2M ELISA measures type II collagen fragments generated by MMPs in cartilage. Briefly, a streptavidin-coated microtiter plate was coated with 4 ng/mL biotinylated peptide. Unbound peptide was washed off and 20 µL 1:2 prediluted serum samples were added, followed by 100 µL of 60 ng/mL peroxidase-labeled monoclonal antibody (MAb-C2M-3C1). The plate was incubated at 4C for 18 hours, washed and developed using 3,3',5,5'-Tetramethylbenzidine (TMB) and stopped with sulfuric acid. The plate was read on a standard plate reader at 450 nm. The C3M ELISA measures type III collagen fragments generated by MMPs in connective tissue. Briefly, a streptavidin-coated microtiter plate was coated with 1.25 ng/mL biotinylated peptide. Unbound peptide was washed off and 20 µL 1:4 prediluted serum samples were added, followed by 100 µL 25 ng/mL peroxidase-labeled monoclonal antibody (MAb-C3M-610T1). The plate was incubated at 20°C for 1 hour, washed and developed using the TMB and stopped with sulfuric acid. The plate was read on a standard plate reader at 450 nm. Technical performances of the assays were assessed according to in-house standard operating procedures and final inspections included tests on detection range, sample stability and linearity, a sample and assay stress test, matrix and interference test, prolonged storage of the assays and normal range tests. In addition, analyte stability was investigated in terms of freeze-thaw, ambient temperatures, prolonged storage, etc. Serum C-reactive protein (CRP) levels were assessed by standard measures. The final inspection results concerning serum testing are summarized in Table 2.

**Table 2:** ELISA technical performance.

	C2M	C3M
*Assay specifications*		
Slope of Standard curve (CV %)	1.10 (6.6)	1.03 (10.8)
IC50, nmol/L (CV %)	0.45 (3.8)	5.22 (11.9)
Intra-assay variation, CV%	6.2	6.7
Inter-assay variation, CV%	7.3	12.1
Lower limit of detection, nmol/L	0.037	0.28
Quantifiable range, nmol/L	0.274–4.36	0.54–42.1
		
*Analyte recovery*		
Serum dilution linearity (analyte recovery)	Neat to 1:4; >82%	1:4 to 1:64; >88%
Freeze-thaw	> 7 cycles	> 10 cycles
On-table stability	>48hours	>48hours
		
*Interference*		
Hemoglobin	<11%	<7%
Lipidimia	<6%	<2%
Biotin	<0.1%	<0.1%
Rheumatoid factor	<0.1%	<0.1%
HAMA	<0.1%	<0.1%
*Biological variation*		
Correlation to age, R, *p-value*	0.083, *ns*	0.13, *ns*
Gender difference, Difference, *p-value*	0.055, *ns*	3.12, *ns*

### Statistics

**Figure 1:**
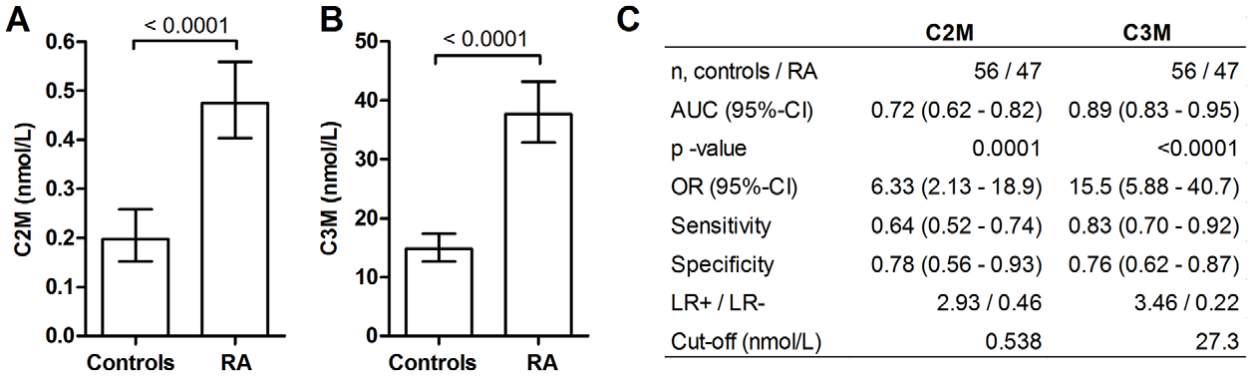
The level of the cartilage degradation and connective tissue inflammation in RA patients (n = 47) and healthy controls (n = 56). A) Cartilage degradation measured by C2M in serum. B) Connective tissue degradation measured by C3M in serum. C) The diagnostic utility depicted as area under the curve (AUC), odds ratio (OR), sensitivity, specificity and likelihood ratio (LR). Cut-off values were set as 1 SD above the mean of the healthy controls. Comparison of RA patients and controls was performed on log transformed data with student’s t-test. Results are shown as geometric mean with 95% CI. Diagnostic utility was calculated by a contingency table applying Fisher’s exact test.

**Figure 2:**
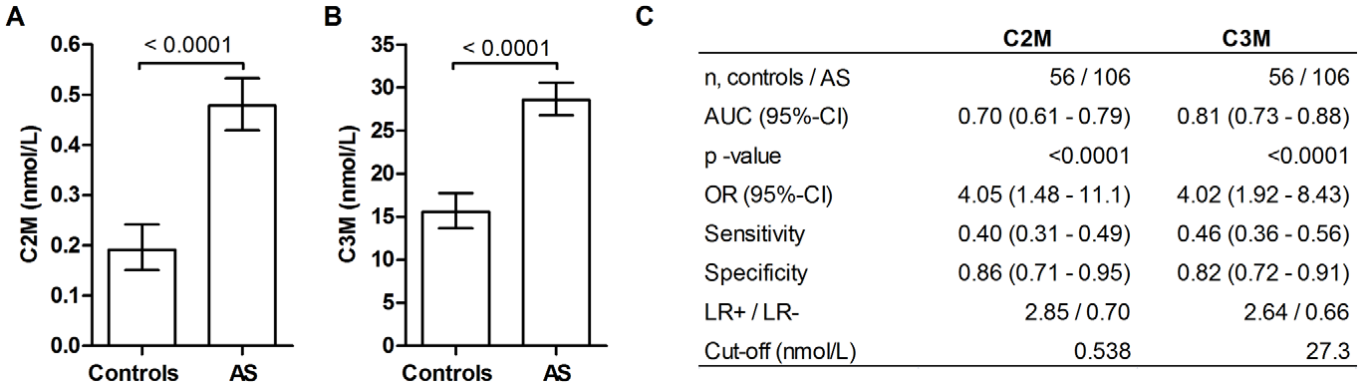
The level of the cartilage degradation and connective tissue inflammation in AS patients (n = 103) and controls (n = 56). A) Cartilage degradation measured by C2M in serum. B) Connective tissue degradation measured by C3M in serum C) The diagnostic utility depicted as area under the curve (AUC), odds ratio (OR), sensitivity, specificity and likelihood ratio (LR). Cut-off values were set as 1 SD above the mean of the healthy controls. Comparison of AS patients and controls was performed on log transformed data with student’s t-test. Results are shown as geometric mean with 95% CI. Diagnostic utility was calculated by a contingency table applying Fisher’s exact test.

Statistical analyses of correlations and logistic regression were performed using MedCalc® version 12 and GraphPad Prism® version 5. The primary objectives were to investigate: i) the serum levels of the two novel biomarkers, C2M and C3M, in RA and AS patients as compared to controls, and ii) whether there was an association between the biomarkers and progression of AS over a 2-year period. Comparison between the log transformed mean levels of the markers was performed using the Student’s t-test (fig. 1, 2). Data was shown as the geometric mean with 95% confidence intervals (95%-CI), which depicts the principal distribution of the biomarker levels. The diagnostic power were investigated by area under the receiver-operator curve (AUROC) giving the AUC with 95%-CI (fig. 1, 2). Using the principle of Z-score normalization, cut-off values for the diagnostic test were set as 1 standard deviation (SD) above the mean of the controls which will include 84% below this cut-off assuming a normal distribution. Using these cut-off values the number of patients below and above the cut-off was counted and put into a 2×2 contingency table. From this the odds ratios (ORs), sensitivity, specificity and likelihood ratios were calculated by Fisher’s exact test (fig. 1, 2).

Univariate correlation analyses between the biomarkers and clinical scores, or between the individual biomarkers, were analyzed by non-parametric Spearman’s test (table 3, 4).

**Table 3:** Univariate analysis for C2M and C3M associations with age, disease duration and RA clinical characteristics; Disease activity score (DAS), health assessment questionnaire (HAQ), erythrocyte sedimentation rate (ESR), high sensitive C-reactive protein (CRP), tender joint count (TJC) and swollen joint count (SJC).

	C2M	C3M
Age	–0.12, *ns*	0.13, *ns*
Disease Duration	0.16, *ns*	0.18, *ns*
DAS	0.09, *ns*	0.54, *p<0.001*
HAQ	0.01, *ns*	0.52, *p<0.001*
ESR	0.05, *ns*	0.60, *p<0.0001*
CRP	–0.06, *ns*	0.59, *p<0.0001*
TJC	–0.02, ns	0.34, *p<0.05*
SJC	–0.10, *ns*	0.18, *ns*
C2M		–0.10, *ns*

Data are shown as Spearman’s correlation coefficient R and *p-value.*

**Table 4:** Univariate analysis for C2M and C3M associations with age, disease duration and AS clinical characteristics; erythrocyte sedimentation rate (ESR), high sensitive C-reactive protein (CRP), mSASSS and BASDAI.

	C2M	C3M
Age	0.06, *ns*	0.17, *ns*
Disease duration	0.01, *ns*	0.14, *ns*
ESR	0.03, *ns*	0.40, *p<0.0001*
CRP	0.06, *ns*	0.55, *p<0.001*
mSASSS	0.02, *ns*	0.32, *p<0.001*
BASDAI	–0.04, *ns*	0.19, *ns*
C2M		0.12, *ns*

Data are shown as Spearman’s correlation coefficient R and p-value.

The prognostic utility was investigated by a decision tree approach by asking following questions: how many AS progressors were we able to select and how many patients were we able to deselect by measuring the two biomarkers in question. The biomarkers for disease progression (NewSynd) was calculated using dichotomized values for the markers by discriminating high biomarker levels by Classification and Regression Tree Analysis (using the 2×2 diagnostic contingency table). Sensitivity (fraction progressors) and specificity (fraction non-progressors) values were produced. Data were considered significant when the P-value was below 0.05.

## Results

### Technical performance of the C2M and C3M ELISAs

The technical performance of the C2M and the C3M ELISAs was assessed before serum levels were measured in patients with RA, AS and healthy controls. Table 2 describes the basic technical characteristics of the two assays, both of which had good technical performance. Intra- and inter-assay coefficients of variation were less than 7% and 13%, respectively. The data analyte recovery, interference (hemoglobin, lipidimia, and biotin, rheumatoid factor (RF) and human anti-mouse antibody (HAMA)) and biological variation are shown in Table 2. None of the markers were significantly correlated to age. There was no gender difference for either marker investigated by Mann-Whitney test.

### The level of cartilage and connective tissue degradation fragments in serum of RA patients

Both serum C2M and C3M levels were significantly higher in RA patients than in healthy controls (*p<0.0001*, fig. 1). Diagnostic utility, as analyzed by ROC and the AUCs, was 72% and 89% for C2M and C3M, respectively (fig. 1C). The odds ratio (OR) for identifying RA patients were 6.3 and 16 for C2M and C3M respectively (fig. 1C). Serum C2M levels were not associated with age, disease duration or any of the clinical outcome scores (Table 3). In contrast, serum C3M levels were highly correlated with disease activity score (DAS), health assessment questionnaire (HAQ), erythrocyte sedimentation rate (ESR), C-reactive protein (CRP), swollen (SJC) and tender joint count (TJC). There was no correlation (table 3) between serum C2M and C3M.

### The level of cartilage and connective tissue degradation in serum of AS patients

Both C2M and C3M levels were significantly higher in AS patients than in healthy controls (fig. 1A and 1B). The diagnostic utility of C2M in discriminating between controls and AS patients was 70% (*p<0.0001*) (Fig. 2C) and thereby similar to that in the RA analysis (Fig. 1C). A high C2M level could correctly identify 40% of the AS patients and 80% of the healthy controls. A high C3M value could correctly identify 46% of the AS patients and 82% of the healthy controls. Thus the sensitivity was markedly lower for AS than for RA. The diagnostic utility of C3M was 81% (*p<0.0001*, Fig. 2C), which was 10% lower than the utility in RA patients (Fig. 1C). The ORs for AS were 3.4 (*p = 0.003*) and 4.7 (*p<0.0001*) for C2M and C3M, respectively (Fig. 2C).

The evaluation of all patients collectively showed C2M correlated with neither of the disease scores; Disease duration, ESR, CRP, mSASSS or BASDAI (table 4). Serum C3M was significantly correlated with ESR (*p<0.0001*) and CRP (*p<0.0001*), as well as with mSASSS (*p = 0.0006*), but was of only borderline significant correlation (*p = 0.054*) with BASDAI (table 4). No correlation was observed between the two biomarkers C2M and C3M when measured in either AS patients, or RA patients (data not shown).

### Clinical predictive utility of measuring serum C2M and C3M

An abnormally high serum C2M level, defined as one SD above the mean of that found in healthy controls, could positively predict 44% of the progressors of AS and exclude 70% of the non-progressors (table 5). High serum C3M levels could positively predict 59% of the progressors and excluded 55% of the non-progressors. A high serum level of both C2M and C3M could predict 80% of the progressors and 61% of the non-progressors.

**Table 5:** Clinical predictive utility of the serum markers.


	High C2M (n = 94)	High C3M (n = 94)	High C2M & high C3M (n = 33)
Cut-offs	>0.538 nmol/L	>27.3 nmol/L	
Sensitivity (%)	44.1 (28.6–61.7)	58.8 (40.7–75.6)	80.0 (51.9–95.7)
Specificity (%)	70.0 (56.8–81.2)	55.0 (41.6–67.9)	61.1 (35.8–82.7)
Positive/Negative Likelihood ratio	1.46/0.80	1.31/0.75	2.06/0.33
Proportion of progressors with a positive test	15/34	20/34	12/15
Proportion of non-progressors with a negative test	42/60	33/60	11/18

Progressors were defined as NewSynd Yes/No over at a two year period.

## Discussion

In the current study we firstly validated the diagnostic utility of the two novel markers, C2M and C3M, in RA and found that the serum levels of the markers were elevated in patients compared with controls with an OR of more than 6. This is expected since RA is characterized by massive joint turnover, which includes cartilage degradation and connective tissue turnover, such as occurs during synovial inflammation. While elevated serum C2M did not seem to be correlated with any disease characteristics in RA, high serum C3M was highly correlated with DAS, HAQ and ESR, as well as TJC. These results support the concept that connective tissue degradation is indeed related to the inflammatory component of RA, whereas cartilage degradation seemed to be partly uncoupled from this component. This suggests that the two biomarkers contribute independent and additive information about the disease pathogenesis and maybe supplementary diagnostic tools for clinical diagnosis. These markers can not compete with diagnostic markers such as RF and CRP, however in stead they can provide additional information on tissue integrity, which may aid in the understanding of disease severity.

Secondly, we investigated whether these joint turnover markers could be used for AS. In contrast to RA, RF and CRP are not applicable diagnostic markers in AS. The present study supports a role of C2M and C3M as potential diagnostic biomarkers in AS. We also saw a strong correlation between connective tissue degradation as measured by serum C3M and the radiographic score, mSASSS. Cartilage degradation as measured by serum C2M levels was likewise elevated in AS patients compared with controls. But C2M did not correlate with the mSASSS. These results are interesting because they indicate that cartilage degradation and soft tissue turnover may provide independent information also in AS.

The first part of the study validated and supports the importance of monitoring connective tissue remodeling in the pathogenesis of rheumatic diseases. The second part of the study evaluated the prognostic potential of the markers. We demonstrated that a combination of these novel markers could identify 80% of the progressors in AS. This combination could be used to select only the likely progressors for clinical trials, and thus reduce the number of patients exposed to treatment in such studies. Furthermore, by identifying likely progressors in an AS population, one could test strategies aimed at early intervention to prevent deterioration of structural damage using anti-inflammatory therapies [21], [22]. Our data also shows that the combination of biomarkers may possess higher prognostic utility than individual biomarkers. We showed that cartilage and soft tissue turnover is indeed increased in AS patients, and that the level of increase is predictive of those who will progress–a potential which CRP does not possess [23].

There are considerable limitations to the information provided by standard clinical and laboratory parameters to guide treatment decisions. Consequently, there has been a particular interest in evaluating biomarkers in AS that reflect disease activity and predict structural progression [5], [24], [25]. Although CRP and ESR are sensitive detectors of disease activity in RA, they are not so in AS, probably because these markers are elevated in only about 50% of AS patients [23]. Unlike in RA, they also correlate poorly with clinical measures of AS, although good correlations have been noted with MRI assessment of inflammation in the spine [26]–[28]. In contrast to RA, CRP and ESR do not appear to predict progression of structural damage in AS although CRP does predict clinical response to anti-TNF therapy in both RA and AS [29], [30].

Cartilage degradation and connective tissue turnover are hallmarks of most arthropathies. We have developed two new serological biomarkers of cartilage degradation (C2M) [12] and soft tissue turnover (C3M) [13], [16]. Both biomarkers are MMP-mediated collagen fragments, which are also called collagen neo-epitopes. Assays for these two biomarkers are specific for the cleavage site in the respective collagens. Hence the assays do not measure full-size collagens, but only the pathogenic fingerprint of that collagen. We have previously demonstrated that serum C2M was associated with increased Kellgreen-Lawrence score in knee osteoarthritis [12], indicating a correlation with cartilage loss and joint deterioration. C3M is derived by degradation of type III collagen, which is a central component of most connective tissues. The turnover of both types II and III collagens is high [31]. There was a strong correlation between CRP and C3M in both RA and AS, which supports the concept that inflammation accelerates the turnover of connective tissue and thereby the release of C3M [14]. This may contribute to the increased levels of these biomarkers in AS which could arise from excessive remodeling of the joint. However, additional contributions may be derived from multiple organs of the body affected by inflammation.

A previous report showed that other markers of extracellular remodeling had limited association with baseline BASDAI and mSASSS [11]. Cartilage oligomeric protein and YKL-40 were both correlated with baseline mSASSS (p<0.05), but they were not associated with 2-year change in mSASSS. Interestingly that study showed a clear association between elevated MMP-3 and radiographic progression. MMP-3 is widely up-regulated by inflammation in the connective tissue surrounding the joints (e.g. synovial tissue). Thus MMP-3 might very well be one of the MMPs responsible for the degradation of type II and type III collagens. It would interesting to investigate the relationship between MMP-3 and type II and type III collagen turnover and potentially combine the markers in an algorithm for predicting inflammatory disease. This kind of investigation will increase our understanding of the role of MMPs in AS and the proteolytical products resulting from MMP activity.

## Conclusions

This is the first study to show the prognostic value of two novel biomarkers of MMP-mediated degradation of types II and III collagen. These two biomarkers, C2M and C3M, might be the best diagnostic [14] and prognostic markers available to date for AS. Further longitudinal study is needed to confirm these preliminary data. We speculate that markers measuring joint tissue turnover and deterioration may assist better understanding of disease pathogenesis and eventually disease severity. In the era of personalized medicine markers that are direct measures of disease status and severity may aid in designing the best treatment for the individual patients.
